# Application of Gut Cell Models for Toxicological and Bioactivity Studies of Functional and Novel Foods

**DOI:** 10.3390/foods1010040

**Published:** 2012-12-13

**Authors:** Martin Trapecar, Avrelija Cencic

**Affiliations:** Department of Biochemistry and Nutrition, University of Maribor, Faculty of Medicine, Slomskov trg 15, Maribor 2000, Slovenia; E-Mail: martin.trapecar@gmail.com

**Keywords:** gut, cell models, risk assessment, toxicology, functional food

## Abstract

The concept of functional and novel foods undoubtedly bears great potential as an asset to human health. However, this very same quest for ever new bioactive ingredients calls for reliable and distinct risk assessment as they may be potentially hazardous to human health. Most of today’s methodologies still rely on decades old routines of animal trials and use of tumor-derived cell lines. Since such methodologies are not in line with the actual processes in the human body and with the 3R (replacement, reduction, refinement) concept, the results are often unreliable and misleading. Therefore, in this paper we propose the utilization of available untransformed small intestinal cell lines derived from human and pig tissue of non-tumor origin and describe several available cell models of the gut that offer a functional, close resemblance with the *in vivo* environment.

## 1. Introduction

Functional food is any food or modified ingredient that can provide a beneficial effect beyond that provided by common nutrients [1]. Since the commercialization of the concept of functional food, immense scientific effort has been put into development and identification of new bioactives and microorganisms that could be used to promote human health, slowly followed by establishment of a regulatory framework to assure consumer safety. The growing markets of functional foods drive the quest for constant innovation which in return gives rise to yet unknown natural or synthetic sources. Such new ingredients with no previously documented attributes bear certain risks which have to be identified and evaluated. 

The evaluation of safety data on functional foods is becoming increasingly important around the world. Its goal is to provide the basis for the assurance of a high level of protection of human health and consumer interest in relation to food [2,3]. In general, toxicology forms the core of risk assessment where safe doses are investigated following the accepted paradigm consisting of: hazard identification; hazard characterization; exposure assessment; and risk characterization [4,5,6]. Risk assessment is mostly triggered by legal requirements or during the process of health claim registration.

Conventional pre-clinical and toxicological practices are often based on decades old methodologies of animal trials and use of tumor-derived cell lines, delivering questionable and unreliable results [7]. *In vitro* models of normal human tissues are therefore strongly gaining importance due to their relevance and wide applicability that ranges from mechanistic studies to risk assessment [8]. Due to reasons stated above and the new legal frames we have accepted the principle of the three Rs (replacement, reduction, refinement) that strives towards new more relevant and efficient test methods [9,10]. There is a need for the scientific community to develop reliable cell culture models that mimic the *in vivo* situation as close as possible.

New insights into human cell biology as well as improvement of culturing techniques are fostering the potential to overcome interspecies differences which are the main cause for the rejection of 92% of all new formulations already in clinical trials [11]. Daneshian *et al.* [12] have in a recent workshop outlined several major examples of interspecies differences between human and animal models. Body size, for example, affects biokinetics and oxidative stress; different species may differ with regard to metabolic enzymes, brain size, developmental speed, and the development of different cancer types—to name a few. Ethical considerations, time consumption and financial input are only additional factors that speak against animal models [13].

In our paper we outline and propose new methodologies such as 3D cell models of the gut that could serve as alternatives for long time overdue practices in risk assessment and toxicological studies of functional foods and other bioactive molecules. 

## 2. Current Approaches in *In Vitro* Toxicology

*In vitro* toxicology as a part of wider risk assessment is the scientific approach identifying harmful effects of xenobiotics or organisms on mammalian cells or bacteria [14]. *In vitro* models can hardly be compared to the complexity of the human body; they therefore mostly relate to specific organ systems that are mimicked by utilizing different cell culture models such as, for example, models of the gut or liver in the case of risk assessment of food. Several approaches have been developed to evaluate potential risks among which the most important are methods to identify direct cytotoxicity and long-term toxicity, genotoxicity, cellular responses and kinetic behavior [15]. 

Cytotoxicity studies are a good starting point that reveals the concentration at which one can observe a necrotic or apoptotic effect. Investigated markers are mostly mitochondrial function, disruption of membranes, changes in cell replication and DNA fragmentation [16,17].

**Figure 1:**
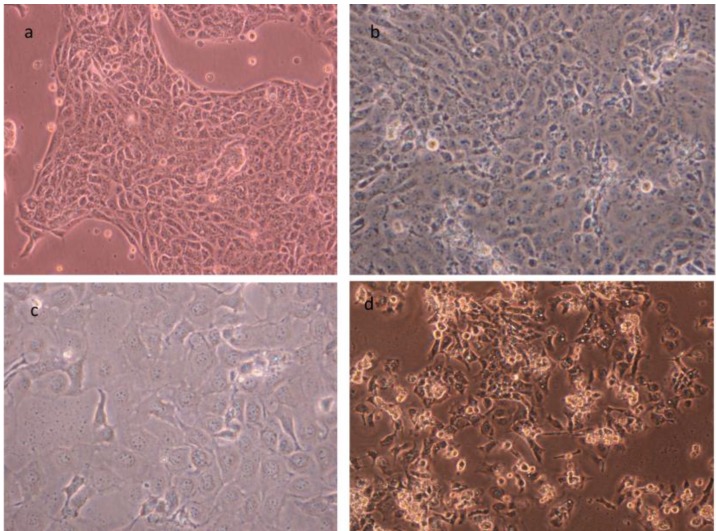
(**a**) H4-1 human small intestinal epithelial cell line; (**b**) PSI-1 pig small intestinal epithelial cell line; (**c**) TLT human monocyte/macrophage cell line; (**d**) Pom2 pig monocyte/macrophage cell line.

With regard to the growing demand for natural compounds and new protective/probiotic strains as components of functional foods, initial cytotoxicity assays help to quickly identify potential harmful effects. It should be noted, however, that only untransformed cell lines can give relevant results, as shown by Trapecar [18]. In a recent study they developed a model appropriate for risk assessment of *Bacillus cereus*. The aim of the study was to create and validate a model that could easily differentiate toxigenic strains from probiotic. This work shows that non-transformed small intestinal epithelial cells PSI (Figure 1) are appropriate for identification of potential toxicity of *B. cereus* strains with a low threshold for risk of enterotoxicity to humans. The same model can be with no doubt applied for assessment of other potential novel probiotic/protective strains. 

As cytotoxicity studies expose a direct, radical impact on cell survival, they do not offer much insight into the underlying mechanisms or long-term effects of exposure. Genotoxicity tests and cellular response studies, on the contrary, have wide-reaching implications. They can be performed on several levels, from genetic expression and translation to a comprehensive metabolomic research. With the “omics” revolution several new possibilities arose, especially with microarrays and MALDI-TOF [13,19]. They enable quick identification of potential toxicity long before any pathological response of the cell. In combination with bioinformatics, reliable prediction models will be developed in the near future [20,21].

Despite the potential of those technologies, their applicability is still very limited, especially since most pathological events take place on the metabolome level. The most important markers of metabolomic cellular stress are oxidative stress, loss of adenosine triphosphate, change in the presence of phase I and II metabolic enzymes, activation of pro-inflammatory cytokines and alteration of proliferation [15,22].

To assess all the risks involved, kinetic profiling of investigated compounds is necessary. ADME (absorption, distribution, metabolism, excretion) studies are performed to determine the bioaccessibility and biotransformation as well as accumulation of bioactives [23,24]. Marques and co-workers [25] have deployed a 3D model of the small intestine to assess toxicity of heavy metals as food pollutants and have obtained relevant results to predict their kinetic behavior. This demonstrates that such models can be easily translated in to food toxicity testing [25]. The subjects of research in such setups are supernatants as well as cell lysates. It is essential to relate toxicodynamic information from *in vitro* systems to real-life situations by transforming concentration-effect relationships to dose-effect relationships, which can be achieved by physiologically based toxicokinetic modeling as outlined in a recent report by Adler [19]. 

The potential risks that novel functional foods may bear cannot be satisfactorily evaluated by single assays. Complex assay batteries and strategies should therefore be developed and standardized according to new developments.

## 3. Cell Cultures

By far the most widely utilized cell cultures in food toxicology are cancer derived or transformed cell lines like CaCo-2 and HT-29. They are valuable in the study of carcinogenic processes but cannot be used to mimic a healthy environment. Despite their human origin they have a phenotype and glycosylation distinct from normal gut epithelia and therefore not provide much advantage over animal models [8,26]. Even more concerning is the wide usage of completely human/gut unrelated cultures like the Chinese hamster ovarian cell line in food research. 

Consideration of phenotype and proper characterization of cell lines is of outmost importance in order to gain relevant results as transformed cell lines suffer from variation in behavior between different laboratories—even with respect to chromosome numbers [27,28].

The wide utilization of carcinogenic/transformed cell lines can probably be attributed to the fact that carcinogenic cell lines can be maintained very easily and were also the first to be stable through higher passages. However, untransformed human as well as animal cell lines are readily available and should be utilized (Table 1).

**Table 1:** Available cell lines and cell models of the pig and human gut [8].

Cell Line/Model	Origin	Type	Status	Species	Supplier
HIEC-6	Small intestine	Epithelia	Normal	Human	University ofSherbrooke ^a^
H4	Small intestinal foetal tissue	Epithelia	Normal	Human	MassachusettsGeneral Hospital ^b^
H4-1	Small intestinal foetal tissue	Epithelia	Normal	Human	BioNutriTech ^c^
PSI-1	Mature small intestine	Epithelia	Normal	Pig	BioNutriTech ^c^
CLAB	Enterocytes	Epithelia	Normal	Pig	BioNutriTech ^c^
Pom 2	Blood	Monocytes	Normal	Pig	BioNutriTech ^c^
TLT	Blood	Monocytes	Normal	Human	BioNutriTech ^c^
Gut 3D model		Functional	Normal	Human	BioNutriTech ^c^
Gut 3D model		Functional	Normal	Pig	BioNutriTech ^c^

^a^ Sherbrooke, Canada; ^b^ Boston, MA, USA; ^c^ Lunel, France.

In light of recent advances in the science of stem cells, intestinal stem cells (ISC) are becoming more and more important also in food and nutrition research. ISC are able to differentiate into absorptive enterocyte, goblet, Paneth and enteroendocrine lineages but require extracellular signals such as Wnt to avoid loss of differentiation [29]. As shown by Ootani *et al.* [29], ISC can be cultured in a 3D matrix environment, developing *in vivo* like properties and structures. 

In such set up they can sustain in culture for over 30 days. If using ISC special care must be taken to validate their functionality once differentiated. ISC in combination with organ like shaped biomaterials as well as incorporation of microfluidics will probably shape the future of *in vitro* food/nutraceutical research once proper validation will be achieved. 

## 4. Available *In Vitro* Cell Models for Risk Assessment and Toxicology Studies of Functional Foods

**Figure 2:**
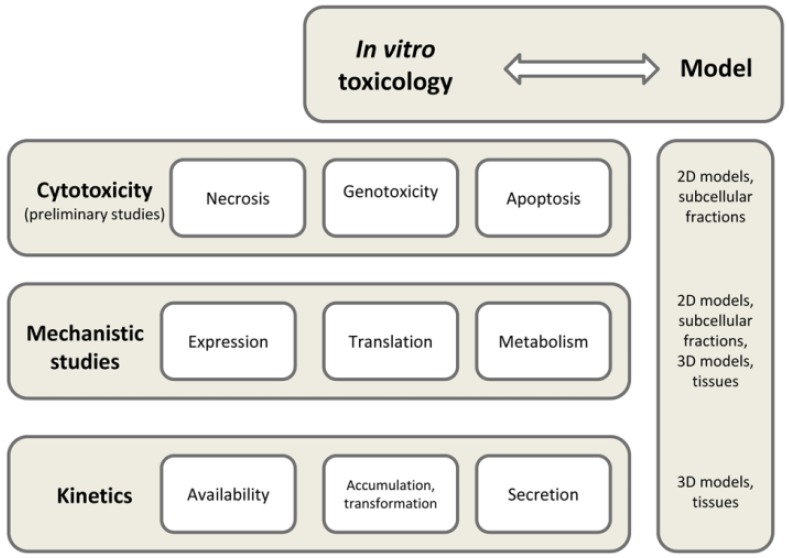
Functional cell models in *in vitro* toxicology.

Cell models are essential tools of toxicology (Figure 2). They are composed of one or more cell cultures, cultivation material like culturing flasks and plates with or without membranes and culturing media. Media strongly differ depending on the needs of individual cultures but most include antibiotics as well as serum if not stated otherwise by protocols. Models are kept in incubators with the presence of 5% CO_2_ at 37 °C. 

Several models have been employed for the purpose of risk assessment: (a) sub-cellular components; (b) cellular systems like primary cell lines, immortalized cell lines, co-cultivation; and (c) tissues [30].

When designing a toxicological study and choosing the right cell model it is essential to address the following major concerns:

- Which cell line has the most relevant phenotype for a designed study?
- Which other organ systems have an influence *in vivo* on our employed system and how can we integrate them in our study?
- Is the model validated and if not, which validated model will be used as comparative control?

Cell lines differing, either due to transformation or inappropriate origin, from the targeting organ may have altered metabolic and morphological properties. It is therefore necessary to choose cell lines with phenotypes as close as possible to the *in vivo* target [31]. At the same time, cells may behave differently when cultivated in an isolated environment missing cell-cell interaction and chemical cross talk [32]. Cultivating cell lines in 3D co-cultures or 3D spheres can overcome this problem.

An essential part of developing cell culture models is their validation and demonstration that their utilization provides equally viable results as animal tests. Several national and supranational institutions like the European Center for the validation of alternative methods (ECVAM), Japanese Center for the validation of alternative methods (JaCVAM) and OECD validate and keep track of new models [12]. 

Depending on the culturing technique, functional cell models can be divided into monolayer or 2D models, multilayer models, and 3D models. Monolayer models are suitable especially for quick determination of cytotoxicity or to monitor specific markers where cell differentiation is not as important, since the objects of observation are single expressed processes. 2D models are usually cultivated on plastic surfaces which do not allow a full differentiation of the cell line [8,31]. 

Such models are suitable for High Throughput Screening (HTS) enabling a fast initial screening of hundreds of compounds and concentrations. 

On the other hand, 3D models with fully differentiated cell lines are more appropriate for mechanistic and kinetic studies. 

**Figure 3:**
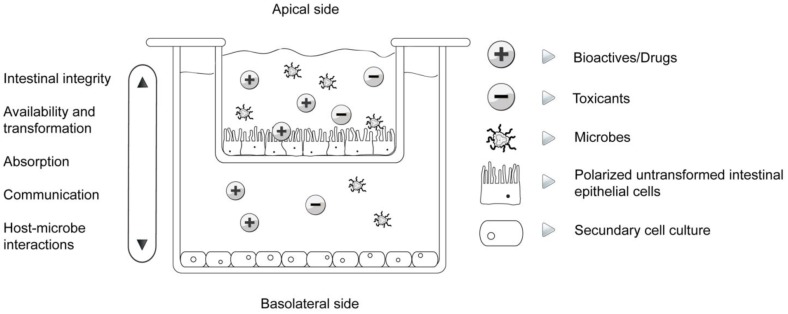
3D functional cell model of the gut.

At this point regarding risk assessment and toxicology studies of functional food, the most interesting alternative is the human 3D co-culture cell model of the gut (Figure 3). 

The 3D co-culture model is composed of a 12 well culturing plate with microporous inserts (Milipore, Billerica, USA), one or more cell cultures and culturing media according to protocols. Inserts divide the wells in to an apical and basal compartment and on the membranes we cultivate untransformed intestinal epithelial cells. Cells grown on membranes are able to differentiate properly through time. The level of differentiation can be monitored by measuring trans-epithelial resistance which is an indicator for the connectedness of cells. Once the epithelial resistance is stable, the model is suitable for experimentation where the influence of investigated compounds on epithelial resistance itself can be an object of research. After differentiation we exchange the culturing media in the apical compartment with media containing the compounds investigated and the basal supernatants with plain media short of investigated bioactives.

Since single cell based assays are not able to adequately represent the complex interplay between different cell types in a specific organ [19], our department has developed several untransformed human and pig small intestinal epithelial as well as human and pig macrophage cell lines that can be co-cultured in a functional 3D model of the gut. 

Mimicking the intestinal barrier, such a model offers immense possibilities from mechanistic to bioaccessibility studies. Moreover, it can be upgraded with an additional culture seeded in the basal compartment such as monocytes/macrophages, skeletal muscle cells, adipocytes or hepatocytes simulating complex systems. 

Previously described untransformed cell lines like H4-1 (human small intestinal epithelial cell line) and PSI-1 (pig small intestinal epithelial cell line) can be cultivated on membranes reaching high *trans*-epithelial resistance and polarization. We have co-cultured them with our human and pig monocyte/macrophage cell lines mimicking the gut and GALT (Figure 1). This and similar models have been successfully used and documented before [8,25,26,33,34]. Table 2 outlines the basic areas in which the model can be utilized. 

**Table 2:** Applicability of the 3D functional cell model of the gut.

Study	Parameter/Implications	References
Trans-epithelial electrical resistance (TEER)	Cell differentiation, connectedness, polarization, intestinal integrity	[8,18,32,35]
Bioaccessibility, absorption and biotransformation	Transition from the apical to the basal compartment and vice versa, cellular absorption, transformation	[8,18,24,25,36,37]
Host-microbe interactions	Attachment, communication, migration, influence on epithelial function, simulation of normal gut microflora, their influence on biotransformation as well as absorption of bioactives	[26,31,37,38,39]
Communication	Cell-cell, cell cross-talk, expression of cytokines, chemokines, nuclear factors, connexins...	[1,8,32,38,40,41]
Immunomodulation	Expression of cytokines and nuclear factors in separated apical and basal compartments, immunoprofiling, pro- and anti-inflammatory orientation	[8,32,37,38,40]
Custom bioassays	Combination of different strategies, combination of different cell lines, HTS integration	[13,30]

Typically, objects of investigation are apical and basal supernatants as well as cell lysates to monitor, for example, signaling molecules, activation factors, compound concentrations and other parameters. 

Basal supernatants can, after the appropriate incubation period, be further transferred on different 2D models for HTS. Toxicological studies with compounds that have passed the simulated intestinal barrier offer much more relevant results than those investigated directly. 

In case of toxicological assessment of foods and supplements, samples can be processed with a simulation of digestive transition prior to applying the samples on cells, as demonstrated by Malvault and co-workers [42].

Several new methods are being developed at this moment, such as integration of biochips, rotation systems and long-exposure static or microfluidic systems [11]. These recent advances in micro engineering redefine our conception of 3D models as they enable true organ like shapes. We therefore distinguish 3D co-culture models using membranes and 3D sphere models that use supportive biomaterials that allow cells to form a lumen. In their recent work, Elamin *et al.* [43] used a base membrane matrix that promoted the development of hollow multicellular spheroid structures by intestinal epithelial cells. In this model cells were able to polarize, form tight junctions and even microvilli.

A step further and what is called the future of pre-clinical research are “organs-on-chips” that integrate microfluidics technologies with living cells cultured within 3D devices created with microfabrication techniques. The base of this technology is lithography and microcontact printing that form defined shapes on a micrometre scale. Not only are different types of cells allowed to align to predefined shapes, also a microfluidic system can be integrated to mimic the flow of body fluids [44]. By extending the microchip architecture “human on a chip”, concepts can be developed where different organ systems can be interconnected. Their potential to predict responses in humans will have profound effects on pre-clinical *in vitro* testing.

## 5. Conclusions

Functional cell models of the gut have an essential role in food risk assessment and toxicology as an alternative to animal studies. They offer a fast and reliable way to characterize new functional foods and bioactives with an in-depth evaluation of underlying mechanisms as well as factors of pathogenicity on a molecular and cellular level. 

Despite the fact that such models lack the complexity of animal models, they have several advantages such as reproducibility of results, controlled environment, and in-depth mechanistic insight. 

With the development of new technologies and cultivation techniques, the identification of various toxicity markers will be possible, enabling establishment of reliable prediction models. From primary cell cultures and carcinogenic cell lines we currently have several untransformed cell lines as well as intestinal stem cells at hand with an appropriate phenotype and metabolic activity. 

We distinguish 2D models appropriate for HTS from 3D models for broader mechanistic studies. Current developments are altering our definition of 3D models as in addition to 3D co-culture models, organ shaped technologies are making its way from lab concepts to industrialization. Gel matrixes allow the development of spheroid structures but their value is limited by major obstacles in regard to probing of transiting molecules. Microengineered chips that also encourage development of higher structures on the other hand enable the incorporation of microfluidics and easy probing techniques. As we do not doubt that in the future human-on-a-chip technologies will be the core of pharmaceutical and food research, at this moment they still lack validation evidence and we are a long way away from seeing their successful commercialization. 

Therefore, it is our opinion that currently the most valuable tools are 3D co-culture models that allow complete polarization, differentiation and crosstalk between different cell types. They can be used to measure bioavailability, biotransformation, immune responses and several other important toxicological parameters and in the same time be linked with HTS 2D systems.

Despite many promising attributes of cell models, their potential has still not been fully exploited. In the future we will have to establish comprehensive toxicological data bases linked with biomarkers, validate new methods, and critically evaluate rooted dogmas. Broader integration of system biology will be necessary to develop organotypic systems that will be closer to the complexity of the human body, especially regarding toxicokinetics. The main goal of risk assessment is to predict and evaluate potential risks to humans. Appropriate models and relevant results are therefore of utmost importance.
